# Screening transferable microsatellite markers across genus *Phalaenopsis* (Orchidaceae)

**DOI:** 10.1186/s40529-017-0200-z

**Published:** 2017-11-15

**Authors:** Ya-Zhu Ko, Huei-Chuan Shih, Chi-Chu Tsai, Hsing-Hua Ho, Pei-Chun Liao, Yu-Chung Chiang

**Affiliations:** 1 Department of Biological Sciences, National Sun Yat-sen University, Kaohsiung, 804 Taiwan; 2 Department of Nursing, Meiho University, Pingtung, 912 Taiwan; 3 Kaohsiung District Agricultural Research and Extension Station, Pingtung, 900 Taiwan; 4 National Pingtung University of Science and Technology, Pingtung, 912 Taiwan; 5 Department of Life Science, National Taiwan Normal University, Taipei, Taiwan; 6 Department of Biomedical Science and Environment Biology, Kaohsiung Medical University, Kaohsiung, Taiwan

**Keywords:** *Phalaenopsis*, Microsatellites, Polymorphism, Transferability

## Abstract

**Background:**

Molecular identification based on microsatellite loci is an important technology to improve the commercial breeding of the moth orchid. There are more than 30,000 cultivars have been enrolled at the Royal Horticultural Society (RHS). In this study, genomic microsatellite primer sets were developed from *Phalaenopsis aphrodite* subsp. *formosana* to further examine the transferability of across 21 *Phalaenopsis* species.

**Methods and results:**

Twenty-eight polymorphic microsatellite markers were obtained using the magnetic bead enrichment method, with high transferability of the 21 species of the genus *Phalaenopsis*, especially in the subgenus *Phalaenopsis*. The 28 newly developed polymorphic microsatellite markers with high polymorphism information content values. The best and second fit grouping (*K*) are inferred as two and four by the *ΔK* evaluation in the assignment test. This result indicates that these microsatellite markers are discernible to subgenus *Phalaenopsis*.

**Conclusions:**

Our results indicate that these new microsatellite markers are useful for delimiting species within genus *Phalaenopsis*. As expected, the genetic relationships between species of subgenus *Phalaenopsis* can be well distinguished based on the assignment test. These molecular markers could apply to assess the paternity of *Phalaenopsis* as well as investigating hybridization among species of genus *Phalaenopsis*.

## Background

The subtropical Taiwan Island that is situated off the southeastern Asian continent has well-suited climate conditions for the growth of orchids. Since the high quality of breeding and micropropagation technology coupled with market demands of the orchid genus *Phalaenopsis* Blume (Orchidaceae), Taiwan has become one of the important exporting countries of orchids in the world (Chen and Chen 2007, 2011; Tang and Chen 2007). The genus *Phalaenopsis* belongs to the family Orchidaceae, subfamily Epidendroideae, tribe Vandeae and subtribe Aeridinae (Dressler 1993), which is often known as moth orchid and comprises approximately 66 species (Christenson 2001). *Phalaenopsis* species is broadly distributed across Himalayas of northern India, South India, Sri Lanka, Southeast China, Taiwan, Indonesia, Thailand, Myanmar, Malaysia, the Philippines, Papua New Guinea and northeastern Australia (Chen and Chen 2011; Christenson 2001). According to the pollinia numbers (Christenson 2001) and molecular evidences (Tsai et al. 2010), *Phalaenopsis* can be divided into five subgenera: the four pollinia clades of subgenera *Proboscidioides*, *Aphyllae*, and *Parishianae* and the two pollinium clades of subgenera *Polychilos* and *Phalaenopsis*. Among these subgenera, the *Polychilos* and *Phalaenopsis* was each subdivided into four sections *Polychilos*, *Fuscatae*, *Amboinenses*, *Zebrinae* and *Phalaenopsis*, *Deliciosae*, *Esmeralda*, *Stauroglottis*, respectively (Dressler 1993; Christenson 2001).

The species of genus *Phalaenopsis* are most popular epiphytic monopodial orchids for their distinctive and varied flowers with the unique structure. Horticultural breeding by hybridization remixed floral characters, such as the colors, shapes, and sizes, to create diversified varieties and cultivars. Based on the high breeding and cultivation techniques for the regulation of light and feeding and the development in interspecific and intergeneric hybrids breeding and polyploidy, improvement of the long-lasting quality of the floral traits made *Phalaenopsis* as one of an important orchid source for cut-flower crop.

There are two indigenous species of *Phalaenopsis* native to Taiwan, the *P. aphrodite* subsp. *formosana* and *P. equestris* (Chen and Wang 1996). Both species were classified as the section *Phalaenopsis* (Christenson 2001). *Phalaenopsis aphrodite* subsp. *formosana*, commonly known as the Taiwan moth orchid, has been widely used as an important breeding hybrids parent, and it is one of the most important progenitors for the traits of modern large and white of floral organs commercial hybrids breeding (Tanaka et al. 2005). *Phalaenopsis equestris* is another important breeding parent for the miniature type of multi-flowers and artificial hybrids with white petals and sepals and a red lip (Men et al. 2003; Tang and Chen 2007).

Recently, intergeneric hybrids between *Phalaenopsis* and *Ascocenda* cultivars were developed to introduce orange color into hybrid cultivars (Liu et al. 2016). However, complex phenotype and long stage of juvenile make the identification of varieties and cultivars of *Phalaenopsis* plants difficult and time consuming. In addition, traditional horticultural breeding technique for new cultivars of *Phalaenopsis* by integrating the morphology, physiological development, and environmental factors as well as their complex interactions makes the breeding consequence unpredictable and uncertain. Molecular markers can provide sensitive and accurate tools for identifying species and cultivars. Therefore, development of highly reliable, rapid, and cheap technique for differentiating and identifying seedlings of species and cultivars of *Phalaenopsis* is necessary and useful for enhancing the efficiency of the breeding. Furthermore, development of molecular markers could apply to paternity analysis, phylogenetic reconstruction, and resolving long-standing issues on *Phalaenopsi*s breeding. Microsatellite markers with characteristics of high level of polymorphism, codominant inheritance and reproducibility (Powell et al. 1996) are useful tools for application in plant genetics and crop breeding, including fruit tree (Chiang et al. 2012; Chiou et al. 2012; Tsai et al. 2013; Lai et al. 2015) and orchid (Tsai et al. 2014, 2015). Compared to previous studies (Sukma 2011; Tsai et al. 2015), we intend to use more microsatellite loci as well as more extensive species testing in this study to enhance the discriminatory power between *Phalaenopsis* genus.

The genome size is small for *P. aphrodite* subsp. *formosana* (Hsiao et al. 2011) and roughly 2.81 pg in diploid genome (Chen et al. 2013), which is suitable for the development of microsatellite markers. Here, the objective of this study was to develop transferable microsatellite markers from *P. aphrodite* subsp. *formosana* using the modified magnetic bead enrichment method. Based on these transferable markers, the molecular identification systems is able to be established for accessing the hybridization and introgression among species of the genus *Phalaenopsis* in future work.

## Materials and methods

### Plant materials

There are 21 species of the genus *Phalaenopsis* comprised of five subgenera used in this study. The taxonomy and nomenclature are followed (Christenson 2001), and specimens information are listed in Table 1. All samples were collected from the plants planted in the greenhouse at the Kaohsiung District Agricultural Improvement Station (KDAIS) in Taiwan by C. C. Tsai. Voucher specimens were deposited in herbarium of the National Museum of Natural Science, Taiwan (TNM).

**Table 1:** Information on geographic distribution, species code and voucher specimens of the genus *Phalaenopsis* used in this study

Classification	Geographical distribution	Code	Source^a^
Subgenus *Proboscidioides* (Rolfe) E. A. Christ.			
*P. lowii* Rchb.f.	Myanmar, and adjacent western Thailand	P4	KDAIS-KC88
Subgenus *Aphyllae* (Sweet) E. A. Christ.			
*P. minus* (Seidenf.) E. A. Christ.	Endemic to Thailand	P11	KDAIS-KC227
*P. braceana* (J. D. Hook.) E. A. Christ.	Bhutan and China	P13	KDAIS-KC289
Subgenus *Parishianae* (Sweet) E. A. Christ.			
*P. parishii* Rchb.f.	Eastern Himalayas, India, Myanmar, and Thailand	P15	KDAIS-KC316
Subgenus *Polychilos* (Breda) E. A. Christ.			
*P. mannii* Rchb.f.	Northeast India, Nepal, and China to Vietnam	P18	KDAIS-KC22
*P. cornu*-*cervi* (Breda) Bl. and Rchb.f.	Northeast India and the Nicobar Islands to Java and Borneo	P2	KDAIS-KC23
*P. kunstleri* J. D. Hook.	Myanmar and Malay Peninsula	P8	KDAIS KC-139
*P. pulchra* (Rchb.f.) Sweet	Endemic to the Philippines (Luzon and Leyte)	P1	KDAIS-KC17
*P. violacea* Witte	Indonesia (Sumatra) and Malaysia (Malay Peninsula)	P9	KDAIS-KC153
*P. micholitzii* Rolfe	Philippines (Mindanao)	P19	KDAIS-KC382
*P. maculata* Rchb.f.	Malaysia (Pahang), East Malaysia (Sabah and Sarawak), and Indonesia (Kalimantan Timur)	P3	KDAIS-KC49
*P. amboinensis* J. J. Sm.	Indonesia (Molucca Archipelago and Sulawesi)	P17	KDAIS-KC157
*P. inscriptiosinensis* Fowlie	Endemic to Indonesia (Sumatra)	P14	KDAIS-KC298
*P. corningiana* Rchb.f.	Borneo (Sarawak and elsewhere on the island)	P16	KDAIS-KC346
Subgenus *Phalaenopsis*			
*P. amabilis* (L.) Blume	Widespread from Sumatra and Java to the southern Philippines, east to New Guinea and Queensland, Australia	P5	KDAIS-KC23
*P. aphrodite* Rchb.f.	Northern Philippines and southeastern Taiwan	PN	KDAIS-KC96
*P. schilleriana* Rchb.f.	Endemic to the Philippines	P10	KDAIS-KC429
*P. chibae* Yukawa	Yukawa endemic to Vietnam	P20	KDAIS-KC488
*P. pulcherrima* (Lindl.) J. J. Sm.	Widespread from northeast India and southern China throughout Indochina to Malaysia (Malay Peninsula), Indonesia (Sumatra), and East Malaysia (Sabah)	P12	KDAIS-KC256
*P. equestris* (Schauer) Rchb.f.	Philippines and Taiwan	P7	KDAIS-KC203
*P. lindenii* Loher	Endemic to the Philippines	P6	KDAIS-KC119

^a^Plant materials were cultivated at the Kaohsiung District Agricultural Improvement Station, Taiwan and voucher specimens were deposited at the herbarium of the National Museum of Natural Science, Taiwan

### Screening, sequencing microsatellite loci, and primer designation

Total DNA was extracted from tissue culture seedlings or young leaves following the procedure by a Plant Genomic DNA Extraction Kit (RBC Bioscience, Taipei, Taiwan). The DNA sample from *P. aphrodite* subsp. *formosana* was screened for microsatellites by digested with the restriction enzyme *Mse*I (Promega, Madison, Wisconsin, USA) and confirmed with 1.5% agarose gel electrophoresis. The digested fragment sizes with a range from 400 to 1000 bps were extracted using agarose gel and then ligated with *Mse*I-adapter pair (5′-TACTCAGGACTCAT-3′ and 5′-GACGATGAGTCCTGAG-3′) using DNA T4 ligase. As the template DNA for the enrichment of the partial genomic library, the ligated products were then used to perform 20 cycles of pre-hybridization PCR amplification in a 20 μL reaction mixture using the adapter specific primer (*Mse*I-N: 5′-GATGAGTCCTGAGTAAN-3′). The PCR mixture contained 20 ng template DNA, 10 pmol *Mse*I-N, 2 μL 10× reaction buffer, 2 mM dNTP mix, 2 mM MgCl_2_, 0.5 U *Taq* DNA polymerase (Promega), and sterile water was added to total volume of 20 μL, with the PCR program of initial denaturation of 94 °C for 5 min, followed by 18 cycles of 30 s at 94 °C, 1 min at 53 °C, 1 min at 72 °C, and a final extension at 72° C for 10 min using a Labnet MultiGene 96-well Gradient Thermal Cycler (Labnet, Edison, New Jersey, USA). The biotinylated oligonucleotide repeat probes (AG)_15_, (AC)_15_, (TCC)_10_, and (TTG)_10_ were used to hybridize with the amplicons at 68 °C for 1 h. The hybridization mixture was then enriched using 1 mg of streptavidin magnesphere paramagnetic particles (Promega) at 42 °C for 2 h and then eluted. Subsequently, DNA fragments containing microsatellites were purified and then amplified by 25-cycle-PCR using purified captured DNA fragments as templates (5 μL), *Mse*I-N (10 pmol), 10× reaction buffer (2 μL), dNTP mix (2 mM), MgCl_2_ (2 mM), 0.5 U *Taq* DNA polymerase (Promega), and supplement sterile water to 20 μL under the amplification conditions described above. The PCR products were purified by the HiYield™ Gel PCR DNA Fragments Extraction Kit (RBC Bioscience) and used for cloning. The purified DNA was ligated into the pGEM^®^-T Easy Vector System (Promega), and used to transformed into *E. coli* DH5α competent cells. The positive clones were randomly selected and used for sequencing. In total, 321 positive colonies were collected and amplified with T7 and SP6 primers and sequenced on an ABI PRISM 3700 DNA Sequencer (Applied Biosystems, Foster City, California, USA). Sequences containing microsatellites were detected using Tandem Repeats Finder version 4.09 (Benson 1999), and primer pairs were designed for microsatellite loci with suitable flanking regions to amplify using FastPCR software version 6.5.94 (Kalendar et al. 2009). Each primer pairs were designed to amplify with a fragment in the range of 100–400 bp.

### Microsatellites PCR amplification

To verify the effectiveness and polymorphisms of 28 microsatellite loci, all primer pairs designed for amplifying these microsatellites were tested using the *P. aphrodite* subsp. *formosana* DNA samples together with the other 20 *Phalaenopsis* species. The optimal annealing temperature was determined using gradient PCR on a Labnet MultiGene 96-well Gradient Thermal Cycler (Labnet). The PCR was carried out in a total reaction volume of 20 μL, in which the PCR reaction mixtures contained 20 ng template DNA, 0.2 μM forward and reverse primers, 2 μL 10× reaction buffer, 2 mM dNTP mix, 2 mM MgCl_2_, and 0.5 U of *Taq* polymerase (Promega). The gradient PCR protocol was set at 94 °C for 5 min, followed by 35 cycles of 94 °C for 40 s, a temperature gradient 50–60 °C for 60 s, 72 °C for 60 s, and a final step of 72 °C for 10 min. Then, the PCR products were assessed using 10% polyacrylamide gel electrophoresis and stained with ethidium bromide and visualized (EtBr) under UV light exposure. The patterns and length of alleles were recorded digitally by Quantity One ver. 4.62 (Bio-Rad Laboratories, Hercules, California, USA).

### Data analyses

In total, 146 repeatable amplicons with length variation were screened from 28 microsatellite primer pairs (Table 2) in 21 species (Table 1). The number and average of amplicons (alleles) per primer pairs and the polymorphism information content (PIC) value of each loci were estimated using Power-Marker version 3.25 (Liu and Muse 2005). The Bayesian clustering method was used to estimate genotyping group information and genetic components for 21 *Phalaenopsis* taxa with the assistance of STRUCTURE ver. 2.3.4 (Pritchard et al. 2000; Falush et al. 2003, 2007). The admixture model (Hubisz et al. 2009) was selected in the Bayesian clustering analysis. The posterior probability of the genetic grouping number (K = 1–21) was estimated using the Markov chain Monte Carlo (MCMC) approach and 10 independent runs with a first 10% discarding (burnin) followed by 5,000,000 MCMC steps for each grouping number. The first-two best grouping numbers were evaluated using ΔK process (Evanno et al. 2005) by STRUCTURE HARVESTER ver. 0.6.8 (Earl and vonHoldt 2012). The graphical display of the results was drawn by DISTRUCT program (Rosenberg 2004).

**Table 2:** Characteristics of the 28 polymorphic microsatellite primers derived from *Phalaenopsis aphrodite* subsp. *formosana*

Locus	Primer sequences (5′–3′)	Repeat motif	Allelic size (bp)	Annealing temperature (°C)	No. of alleles	PIC
PA5-1	F: TCCCATTATCACTCCCTCAC	(TC)_14_	140–164	59	4	0.67
	R: GGTTAGAGATATAGGGAGAG					
PA5-2	F: CTCTCTTTCCTTCTCACCTC	(TC)_10_	98–104	58	4	0.61
	R: AAGATAGAGGGAGAGAGTGG					
PA7	F: CTCTGCTTCTCACCTTTCAC	(TC)_12_	116–264	56	3	0.55
	R: GGACAGAAAGTGAGAGAGAG					
PA10	F: TCTTCAGTCCCTCACTCATC	(CT)_14_	132–152	58	7	0.75
	R: ACAAAGCGGTGGAGAATATG					
PA11	F: ATCTATTGCTCTTTGTCCTC	(CT)_42_	214–216	55	2	0.38
	R: TAGCAAAGAGATGCTGAAGG					
PA14	F: TTTTCACTCTCCCTCCATCC	(CT)_21_	182–186	52	3	0.55
	R: GATGTAGAGAATGAGGGAGC					
PA15	F: TCTCCTACTCCCTCTATCTCA	(CT)_25_	306–310	56	3	0.55
	R: CTTGAAAGGCAGAGAGATAG					
PA19	F: TCTCCCTATATCTCTGCATC	(CTCC)_4_	154–158	51	3	0.58
	R: TGGAAAGAGAAAGGTTCAGG					
PA21	F: TCTCTCACTTTGTCACTCGC	(CT)_14_	134–146	57	6	0.77
	R: AAAGGGAAGTAGGGAAGGAG					
PA24	F: TTGATCTCTCTGGCACCCAC	(TC)_36_	216–224	55	3	0.59
	R: AAGAGAGAGTTAGTTGGAGAT					
PA25-1	F: ACCCACTTTCTCCTATCTCC	(CT)_20_	176–202	58	5	0.64
	R: GATGAAAGAGAGTGAGAGCG					
PA25-2	F: TCTCCCTCTCTTTACCACTC	(CT)_12_	92–267	58	6	0.73
	R: GTGAGAGAGATAGAGTGAGC					
PA32-1	F: CTCTTCCTGCTTTTCCTAGG	(CT)_25_	148–222	57	8	0.83
	R: AAGAGGGTGTGAGGAAGAGG					
PA32-2	F: TCTCTCACTACTCTATCTTG	(CT)_18_	140–152	54	5	0.73
	R: GAGAAGATAGAAAGAGTGAG					
PA36	F: CTCCACTTTATCTCTCTACC	(TC)_39_	220–250	55	4	0.67
	R: ATTGAGCGAGATAAAACTAG					
PA37	F: TTTACCTCTTTTGCTAGCTC	(TC)_23_	226–234	50	4	0.67
	R: AAGAGAAAGGGAAGGAGAGC					
PA38	F: CTCTCTCACTCTATTACTCC	(CT)_32_	224–384	54	9	0.84
	R: AGCTAGATAGAGGGAGAAAG					
PA40	F: GAGCAACATTCACTAGAGAG	(CA)_14_	258–320	56	6	0.79
	R: CTGGCAAAGCTTTGAGAAGG					
PA41	F: GAGGAGAAATAATGATTCCG	(AG)_12_	138–140	50	2	0.38
	R: AGACACTCTCACACACTTTC					
PA63	F: TTCATTCCATCTACCCCATC	(CT)_8_	130–136	55	3	0.59
	R: GATAGAAAGACTAGAGTAGG					
PA64	F: CTCTCCTTTTTCTTATCTTTCAC	(CT)_94_	248–296	55	3	0.55
	R: TAGAGAGATAGAGGGCAAGC					
PA74-1	F: AATGACCTCTCTGCTCTCTC	(TC)_28_	172–306	50	3	0.59
	R: GCAAGAGAAGTTGTGGGATGG					
PA74-2	F: CATCCCACAACTTCTCTTGC	(CT)_13_	122–134	55	5	0.58
	R: AGTGCTCAAGCGAGTTAGAGAC					
PA83-1	F: CCCTCTTTCTCTCATTGTCC	(TC)_9_	190–198	54	2	0.35
	R: GGGACAGAGTGCATAAGATG					
PA83-2	F: CCTTATCTCTTCTCTCTACC	(TC)_36_	168–210	50	12	0.87
	R: AGAAAGGAAGGGTAGGAGAG					
PA100-1	F: TCCCTCTATTTTAGACACCC	(TC)_11_	132–136	52	2	0.35
	R: GGAGAAAGAGCAAGACAGTG					
PA100-2	F: TCTCCATCCGTTAGCCTCTC	(CT)_16_	128–136	59	5	0.73
	R: GGGTAGGCAGAGAGAGTGAT					
PA101	F: CCCACTCACACTCTATCTTC	(TC)_11_	126–138	55	7	0.63
	R: AGGGTCAAACAGAATGAAGG					
				Average	4.57	0.63

## Results and discussion

All of 21 *Phalaenopsis* species reveal either zero, one or two PCR amplicons in each of 28 microsatellite loci. One or two PCR amplicons per locus represent homozygotes or heterozygotes, and no amplicon indicate lacking this homologous microsatellite locus (Table 2). The genome size of *Phalaenopsis aphrodite* subsp. *formosana* detected by flow cytometry reveals roughly 2.81 pg in diploid genome (Chen et al. 2013) and all diploid species of *Phalaenopsis* have 38 chromosome number (Christenson 2001). These related studies and our current results indicate that 21 *Phalaenopsis* taxa studied are diploid plants, except the *P. lowii* and *P. minus* are not listed in the study of Chen et al. (Christenson 2001; Chen et al. 2013).

In total, 146 amplicons (alleles) were identified by 28 microsatellite primer pairs across 21 native *Phalaenopsis* species, and the number of amplicons per primer pairs ranged from 2 to 12, with an average of 5.21 (Table 2). The cross-species amplification test for the 20 other species was conducted using 28 microsatellite primers developed by *P. aphrodite* subsp. *formosana*, and the species of *P. amabilis* (L.) Blume, *P. schilleriana* Rchb.f, *P. chibae*, *P. equestris* (Schauer) Rchb.f. and *P. lindenii* Loher have higher transferable loci. The above mentioned four species with *P. aphrodite* subsp. *formosana* are all classified under the genus *Phalaenopsis*. The microsatellite primers could be successfully transferable to an average of 6.21 species [range from two (PA7, PA11 and PA41) to 20 (PA101) species] (Table 3). Due to the high transferability to species of the subgenus *Phalaenopsis*, these newly developed microsatellite primers are able to apply to establish a standard molecular identification operating system in *Phalaenopsis*.

**Table 3:** The result of the 28 polymorphic microsatellite loci isolated from *Phalaenopsis aphrodite* subsp. *formosana* test on 21 samples

Locus	PN	P1	P2	P3	P4	P5	P6	P7	P8	P9	P10	P11	P12	P13	P14	P15	P16	P17	P18	P19	P20	IS
PA5-1	*O*	–	–	*O*	–	*O*	*O*	*O*	*O*	–	–	–	–	–	–	–	–	–	–	–	–	6
PA5-2	*O*	–	–	–	–	*O*	*O*	–	*O*	–	*E*	–	–	–	–	–	–	–	–	–	–	5
PA7	*E*	–	–	–	–	–	*O*	–	–	–	–	–	–	–	–	–	–	–	–	–	–	2
PA10	*O*	*O*	*O*	*E*	–	*E*	*O*	*E*	–	–	*O*	–	*O*	–	–	–	*O*	–	*O*	–	*O*	12
PA11	*O*	–	–	–	–	–	–	–	–	–	*O*	–	–	–	–	–	–	–	–	–	–	2
PA14	*O*	–	–	–	–	–	*O*	*O*	–	–	*O*	–	–	–	–	–	–	–	–	–	–	4
PA15	*O*	–	–	–	–	–	–	*O*	–	–	*O*	–	–	–	–	–	–	–	–	–	*O*	4
PA19	*O*	–	–	–	–	*O*	*O*	*O*	*O*	–	*O*	–	–	–	*O*	–	–	–	–	–	*O*	8
PA21	*O*	–	*O*	*O*	*O*	*O*	*O*	*O*	*O*	–	*E*	*O*	–	–	*O*	–	–	–	–	–	–	11
PA24	*O*	–	–	–	–	*O*	*O*	–	–	–	–	–	–	–	–	–	–	–	–	–	–	3
PA25-1	*O*	–	–	–	–	*O*	*O*	*O*	–	–	–	–	–	–	–	*O*	*O*	–	*E*	–	*O*	8
PA25-2	*O*	–	–	–	–	–	–	*E*	–	–	*O*	*E*	–	–	–	–	–	–	*E*	–	–	5
PA32-1	*E*	–	–	–	–	–	*O*	*O*	*O*	–	*O*	–	–	–	–	–	*O*	–	–	*O*	*O*	8
PA32-2	*O*	–	–	–	–	–	*O*	*E*	–	–	–	–	–	–	–	*O*	–	–	*O*	–	*E*	6
PA36	*O*	–	–	–	–	–	–	–	–	–	*O*	–	–	–	*O*	–	–	–	–	*O*	*O*	5
PA37	*O*	–	–	–	–	–	*E*	*O*	–	–	–	–	–	–	–	–	–	–	–	–	–	3
PA38	*O*	–	–	–	*O*	–	*O*	*O*	*O*	–	*E*	*O*	*O*	–	–	–	–	–	–	–	*E*	9
PA40	*E*	–	–	–	*O*	–	–	*O*	*E*	–	–	–	–	–	–	–	–	–	–	–	–	4
PA41	*O*	–	–	–	–	–	–	–	–	–	*O*	–	–	–	–	–	–	–	–	–	–	2
PA63	*O*	–	–	–	–	*O*	–	*O*	–	–	–	–	–	–	–	–	–	–	–	–	–	3
PA64	*O*	–	–	–	–	*O*	–	*O*	–	–	–	–	–	–	–	*E*	–	–	–	–	–	4
PA74-1	*O*	*O*	–	–	–	–	*O*	–	–	–	–	–	–	–	–	–	–	–	–	–	–	2
PA74-2	*O*	–	–	–	–	*O*	*O*	*O*	–	–	*E*	–	*E*	–	–	–	–	–	–	–	–	6
PA83-1	*O*	–	–	–	–	–	–	–	–	–	–	–	–	–	–	–	–	–	–	*O*	*O*	3
PA83-2	*E*	*O*	*O*	–	*E*	–	*O*	*O*	*O*	–	*E*	*O*	*O*	–	–	*E*	–	–	*O*	*E*	*E*	14
PA100-1	*O*	–	–	–	–	*O*	–	*O*	*O*	–	–	–	–	–	–	–	–	–	*O*	–	*O*	6
PA100-2	*O*	–	–	–	–	*O*	*O*	*O*	*O*	*O*	*O*	*O*	–	–	–	–	–	–	*O*	–	–	9
PA101	*O*	*O*	*O*	*O*	*O*	*O*	*E*	*E*	*O*	*O*	*O*	*O*	*O*	*O*	*O*	*O*	*O*	*O*	*O*	–	*O*	20
IL	28	4	4	4	6	13	18	20	11	2	16	5	5	1	4	5	4	1	8	4	12	

*IS* the number of successful amplified species, *IL* the number of successful amplification primer, *O* homozygote, *E* heterozygote

The allelic polymorphism information content (PIC) values reflect the extent of allele diversity among the species, the PIC values in the present study ranged from 0.38 to 0.87, with an average of 0.63 (Table 4). Previous studies showed that the PIC values ranged from 0.1754 to 0.6740 (Sukma 2011) and 0 to 0.682 (Tsai et al. 2015) for the genomic microsatellite loci and EST-SSR of *Phalaenopsis* species, respectively. Thus, the PIC value in our study is greater than previous studies on *Phalaenopsis.* This PIC result is consistent with genomic microsatellite studies in *Scutellaria austrotaiwanensis* (Hsu et al. 2009), mango (Chiang et al. 2012), and Indian jujube (Chiou et al. 2012).

**Table 4:** Proportion of individuals of each pre-defined population in each of the 2 and 4 clusters

	*K* = 2		*K* = 4				Subgenus	Section
	Composition 1	Composition 2	Composition 1	Composition 2	Composition 3	Composition 4		
*P. aphrodite*	*0.995*	0.005	*0.625*	0.371	0.001	0.002	*Phalaenopsis*	*Phalaenopsis*
*P. amabilis*	*0.689*	0.311	*0.572*	0.164	0.238	0.027	*Phalaenopsis*	*Phalaenopsis*
*P. schilleriana*	*0.904*	0.096	*0.800*	0.155	0.028	0.017	*Phalaenopsis*	*Phalaenopsis*
*P. equestris*	*0.988*	0.012	*0.824*	0.145	0.003	0.029	*Phalaenopsis*	*Stauroglottis*
*P. lindenii*	*0.974*	0.026	0.231	*0.478*	0.002	0.288	*Phalaenopsis*	*Stauroglottis*
*P. chibae*	*0.529*	0.471	0.106	0.133	0.116	*0.645*	*Phalaenopsis*	*Deliciosae*
*P. pulcherrima*	0.117	*0.883*	0.047	0.028	*0.894*	0.031	*Phalaenopsis*	*Esmeralda*
*P. cornu*-*cervi*	0.009	*0.991*	0.002	0.002	*0.992*	0.004	*Polychilos*	*Polychilos*
*P. mannii*	0.313	*0.687*	0.183	0.054	*0.643*	0.121	*Polychilos*	*Polychilos*
*P. kunsteri*	*0.568*	0.432	*0.488*	0.111	0.391	0.01	*Polychilos*	*Fuscatae*
*P. violacea*	0.016	*0.984*	0.003	0.002	*0.992*	0.003	*Polychilos*	*Amboinenses*
*P. maculata*	0.047	*0.953*	0.01	0.012	*0.958*	0.02	*Polychilos*	*Amboinenses*
*P. pulchra*	0.032	*0.968*	0.007	0.004	*0.983*	0.006	*Polychilos*	*Amboinenses*
*P. micholitzii*	0.239	*0.761*	0.036	0.058	*0.643*	0.263	*Polychilos*	*Amboinenses*
*P. amboinensis*	0.007	*0.993*	0.002	0.002	*0.994*	0.002	*Polychilos*	*Amboinenses*
*P. inscriptiosinensis*	0.054	*0.946*	0.01	0.013	*0.922*	0.055	*Polychilos*	*Zebrinae*
*P. corningiana*	0.028	*0.972*	0.003	0.024	*0.859*	0.114	*Polychilos*	*Zebrinae*
*P. lowii*	0.214	*0.786*	0.063	0.078	*0.815*	0.043	*Proboscidioides*	*Proboscidioides*
*P. parishii*	0.147	*0.853*	0.072	0.019	*0.887*	0.022	*Parishianae*	*Parishianae*
*P. minnii*	0.081	*0.919*	0.029	0.008	*0.957*	0.006	*Aphyllae*	*Aphyllae*
*P. braceana*	0.007	*0.993*	0.002	0.002	*0.994*	0.002	*Aphyllae*	*Aphyllae*

Italic values indicate major component of the species

**Fig. 1:**
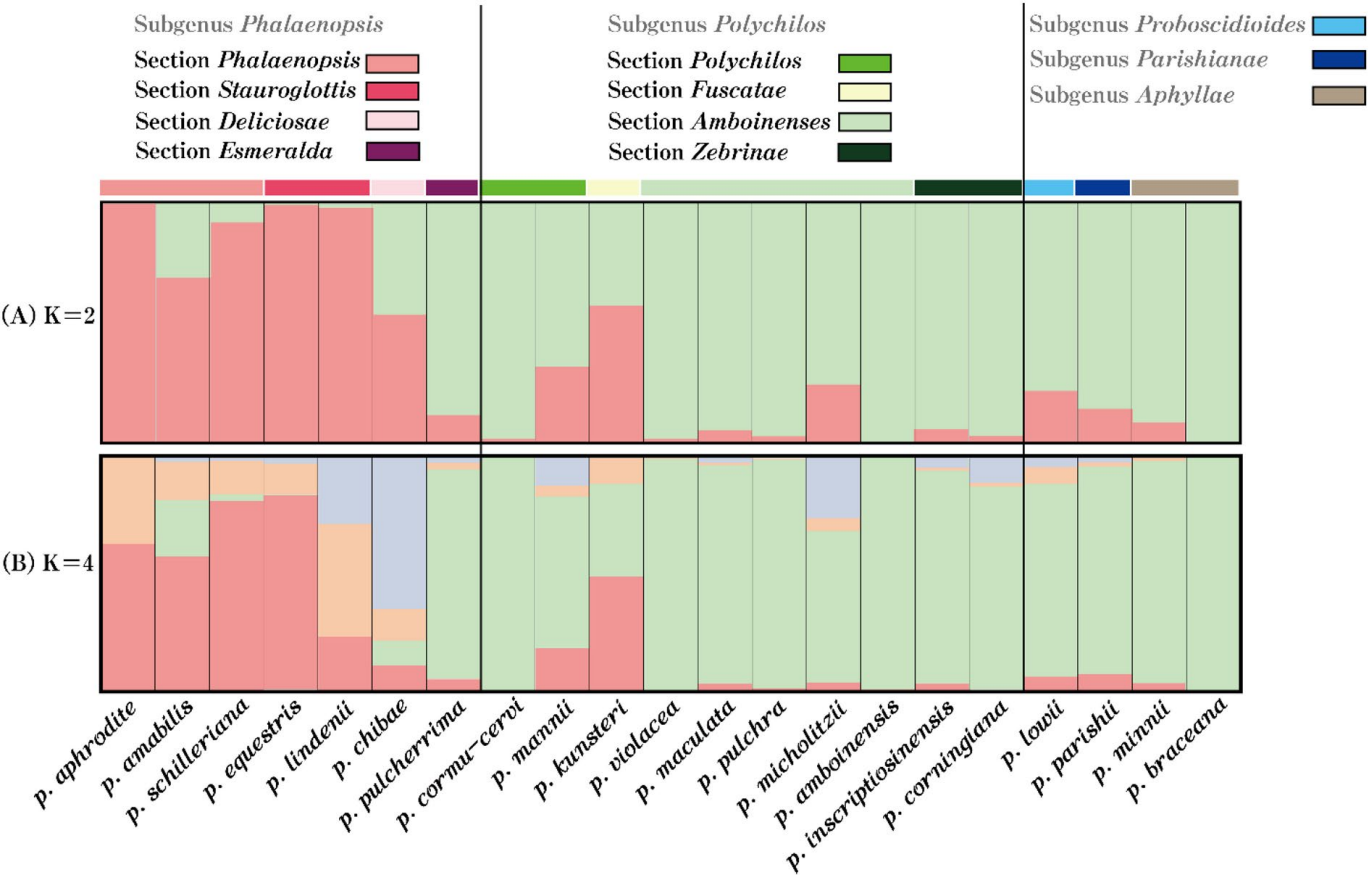
Genotypic group structure of the genus *Phalaenopsis* used in this study in *K* = 2 (**A**) and 4 (**B**)

For genetically delimiting 21 species of the genus *Phalaenopsis*, a model-based Bayesian clustering algorithm was performed in STRUCTURE 2.3.4. The result showed that the first two best clustering numbers are *K* = 2 and *K* = 4 (Table 4). The Δ*K* was 96.55 and 2.31 when *K* = 2 and *K* = 4 in the Bayesian clustering analysis, respectively. Under *K* = 2, most species of the subgenus *Phalaenopsis* were assigned to the same cluster with high percent of Component 1 (pink segment in Fig. 1A) except *P. pulcherrima* that is genetically assigned to sections *Esmeralda* (subgenus *Polychilos*). The subgenus *Proboscidioides*, *Aphyllae*, and *Parishianae*, and *Polychilos* were consigned to the cluster with high percent of Component 2 (green segment in Fig. 1A) except *P. kunstleri* belonging to subgenus *Polychilos* which revealed an admixture genetic composition (56.8% of Component 1 and 43.2% of Component 2) (Table 4). When *K* = *4*, Component 1 of *K* = 2 was divided into three components, 1a (pink segment in Fig. 1B), 1b (orange segment in Fig. 1B), and 1c (purple segment in Fig. 1B) (Table 4). Under *K* = 4, sections *Deliciosae* and *Esmeralda* can be divided into different clusters, which are grouped together when *K* = 4. Two sections *Phalaenopsis* and *Stauroglottis* of subgenus *Phalaenopsis* were grouped together with high genetic similarity (Table 4 and Fig. 1B). In addition, section *Fuscatae* of subgenus *Polychilos* was genetically assigned to the subgenus *Phalaenopsis* cluster based on both section *Fuscatae* of subgenus *Polychilos* belong to pink segment group with more than 50% proportion of Component 1 (see Fig. 1A, B and Table 4). The assignment test by Bayesian clustering analysis reveals similar result with molecular phylogeny patterns described by Tsai et al. (2005). The Bayesian clustering analysis based on EST-SSR loci could not get high resolution between either subgenus or sections within subgenus (Tsai et al. 2015). Compare to EST-SSR results published by Tsai et al. (2015), these newly developed genomic microsatellite loci have higher resolution than EST-SSR loci when study on native moth orchids.

## Conclusions

The *Phalaenopsis* species are important genetic resources for the breeding of hybrids in the horticultural market. The molecular identification markers are an important technology for breeder to improve the commercial cultivars. In this study, we developed 28 primer sets for the polymorphic microsatellite loci of *Phalaenopsis aphrodite* subsp. *formosana*, which are highly transferable among related species of the genus *Phalaenopsis*. Based on these transferable markers, delimitations between subgenera and between sections inferred by the Bayesian clustering analysis indicate that these SSR markers reveal high taxonomic resolution for paternity and hybridization application among genus *Phalaenopsis*. In this study, we provided useful and cheap DNA barcoding markers for molecular breeding.
